# A Review of the Preventive Strategies for Venous Thromboembolism in Hospitalized Patients

**DOI:** 10.7759/cureus.48421

**Published:** 2023-11-07

**Authors:** Chidera Onwuzo, John Olukorode, Walid Sange, Shrushti Jayesh Tanna, Osadebamwen W Osaghae, Abdulraheem Hassan, Heritage Kristilere, Dolapo A Orimoloye, Olutomiwa Omokore, Busayo Ganiyu, Temiloluwa Fayemi, Ehizobhen Addeh

**Affiliations:** 1 Internal Medicine, Benjamin S. Carson College of Health and Medical Sciences, Ilishan-Remo, NGA; 2 Internal Medicine, K. J. Somaiya Medical College & Research Centre, Mumbai, IND; 3 Internal Medicine, Smt. Kashibai Navale Medical College and General Hospital, Pune, IND; 4 Internal Medicine, College of Medicine, University of Lagos, Lagos, NGA

**Keywords:** deep vein thrombosis (dvt), thromboprophylaxis, patient-centered method, multimodal approach, venous thromboembolism (vte)

## Abstract

This comprehensive review introduces the critical issue of venous thromboembolism (VTE), emphasizing its prevalence, particularly in developed countries, and its role as a leading cause of preventable deaths. It discusses the components of VTE, encompassing deep vein thrombosis (DVT) and pulmonary embolism (PE), with a focus on the clinical challenges of diagnosing silent VTE in hospitalized patients.

The review underscores the shocking statistics associated with VTE, including its impact on patient mortality, especially in medically treated, acutely ill patients. Despite the availability of evidence-based guidelines recommending VTE prophylaxis, there is a significant gap in implementation, making it a leading cause of unexpected hospital deaths.

Additionally, the review outlines the multifaceted nature of VTE risk factors, ranging from transient to persistent and provoked to unprovoked, providing a comprehensive understanding of patient-specific considerations. The latter part of the review delves into the impact of VTE on patient health outcomes, revealing its adverse effects on survival, recurrence rates, and psychosocial well-being. Furthermore, it explores various preventive measures, including pharmacological and mechanical options, and their effectiveness, highlighting the importance of a multimodal approach. The review also touches on the challenges of guideline adherence and patient-centered considerations in VTE prevention.

## Introduction and background

Venous thromboembolism (VTE) is a significant global health challenge, being the leading cause of preventable deaths in developed countries [1]. VTE comprises deep vein thrombosis (DVT), where blood clots form in deep veins, often in the legs or pelvis, and pulmonary embolism (PE), a severe outcome when these clots break free, traveling through the heart to block the pulmonary arteries [2].

Startling statistics show that VTE affects about 900,000 patients yearly, contributing to 100,000 to 300,000 deaths in the United States alone. VTE can be clinically silent, making it hard to diagnose for many hospitalized individuals [1]. Placebo-controlled trials have revealed subclinical VTE rates of 5% to 28% among acutely ill medical patients, with potential reductions of half to two-thirds through proper prophylaxis [3].

Tragically, a significant portion of fatal pulmonary embolism cases happens in medically treated, acutely ill patients. Current guidelines, based on extensive trials, recommend broader VTE prophylaxis. Yet, despite strong evidence, VTE prophylaxis remains underused, making it the leading cause of unexpected hospital deaths [3].

Despite awareness efforts, over 40% of at-risk hospitalized patients don't receive pharmacological VTE prophylaxis. This highlights the limitations of current approaches, mainly relying on ongoing education. A more effective VTE prevention program could save almost half a million lives yearly in the United States and European Union nations combined [1].

This review emphasizes understanding VTE and its prevention. Here we look to explore various preventive measures, including pharmacological and mechanical options, and their effectiveness. We will also shed light on existing guidelines and the challenges of adherence. Addressing this critical issue is key to improving patient outcomes and reducing the VTE burden in healthcare.

Understanding VTE

Rudolph Virchow's triad outlines three main factors leading to venous thrombosis: venous stasis, blood hypercoagulability, and vascular wall injury. Venous stasis may be a product of immobility. Several hematologic abnormalities of coagulation factors or natural anticoagulants increase blood hypercoagulability and thrombotic risk. Vascular wall injury promotes the circulation of coagulation enzymes and cofactors [2].

Pathological thrombosis in veins or arteries occurs due to coagulation either in the absence of vascular injury or at injury sites. Most deep vein thrombi form on intact endothelium, especially within valve pockets, where lower blood oxygen levels and blood stasis occur. In pulmonary circulation, thrombi can arise in situ or as emboli when venous thrombi become unstable, detach, embolize to the lungs, and obstruct pulmonary circulation [4].

Risk factors for VTE can be provoked or unprovoked, and they can be transient or persistent. Understanding their nature is crucial for determining appropriate anticoagulation therapy. Thrombosis linked to transient risk factors carries a lower recurrence risk after stopping therapy. In contrast, thrombosis provoked by progressive and persistent risk factors poses a higher recurrence risk upon discontinuation of anticoagulation [2].

Examples of persistent provoked risk factors include active cancer, congestive heart failure (CHF), obesity, and varicose veins. Transient-provoked risk factors involve situations like extended bed rest, estrogen therapy, surgery, pregnancy, or certain injuries associated with immobility [2]. Some other risk factors include hereditary risk factors, such as deficiencies in natural anticoagulants like protein C, protein S, and antithrombin; these significantly increase the risk of VTE [2].

The impact of VTE on patient health outcomes

VTE has adverse impacts on patients’ health outcomes. To begin with, patients with VTE have reduced survival compared to populations of similar age, sex, and ethnic distribution. For almost one-quarter of pulmonary embolism patients, the initial clinical presentation is sudden death [5]. In other words, VTE accounts for a significant number of premature deaths among people who suffer from it [6]. Survival is worse when PE occurs after DVT than when DVT occurs alone [5]. 

A study done by Spencer et al. in 2008 revealed that DVT and/or PE are also associated with recurrence and major bleeding episodes. The study also showed an association between initial standalone DVT and subsequent PE. Among patients who had isolated DVT, 5.6% had subsequent PE, 19% had recurrent VTE, 12.8% experienced a major bleeding episode, and 36.0% died during the follow-up period [7].

A qualitative study done in the United Kingdom explored the long-term psychosocial impacts VTE patients suffer. These included negative feelings like sadness and a loss of independence but of worthy note is the ’Post-Thrombotic Panic’, which signifies anxiety felt by patients because of a fear of recurrence. These feelings are usually triggered by sensations associated with their initial episode of VTE such as breathlessness. This then posed the challenge of differentiating a recurrence from a panic attack. Overall, the symptoms can be debilitating for patients [8].

## Review

Pharmacological preventive measures

VTE, including conditions like PE and DVT, poses a substantial risk to acutely ill, immobilized medical patients. Preventing VTE is crucial in avoiding potentially life-threatening complications. This can be achieved by pharmacological methods, mechanical methods, or a combination of both. Here, we focus on pharmacological approaches for VTE prophylaxis.

Pharmacological prevention methods involve using drugs like low-dose unfractionated heparin (LDUH), low-molecular-weight heparins (LMWHs), direct oral anticoagulants (DOACs), and fondaparinux.

LMHWs exert their effects primarily by reducing the activity of factor Xa and, to a lesser extent, factor IIa (thrombin) through antithrombin activation, effectively inhibiting clot formation. They include drugs like dalteparin, enoxaparin, reviparin, and others. The PREVENT trial aimed to evaluate dalteparin sodium's potential in lowering VTE risk among patients aged 40 and older with acute medical conditions during hospitalization. This trial demonstrated that dalteparin, administered at 5000 IU per day, effectively reduces clinically significant VTE without increasing the bleeding risk in medically ill patients hospitalized for a minimum of four days, with ≤3 days of prior immobilization. These findings endorse the routine use of dalteparin for thromboprophylaxis in this patient cohort [9]. Research, such as the MEDENOX trial, has demonstrated that prophylactic treatment using 40 mg of subcutaneous enoxaparin, another LMWH, per day is safe and effective for patients with acute medical conditions to decrease the risk of VTE [10].

Unfractionated heparin, another commonly used drug for VTE prevention, functions by activating antithrombin, leading to decreased action primarily of factors IIa (thrombin) and Xa. A study by Kahn et al. suggests that LMWH and UFH (unfractionated heparin) appear to have similar effects in reducing thrombosis among acutely ill hospitalized medical patients. However, there remains a degree of uncertainty regarding the relative effectiveness of these treatments. While LMWH offers a potential advantage of less bleeding, the difference may be minimal [11]. UFH is preferred in patients with renal failure due to its shorter half-life, and therefore faster clearance, reducing the risk of bleeding [12]. 

DOACs especially apixaban and rivaroxaban have shown efficacy in prophylaxis of VTE in cancer patients [13]. The VALERIA (Venous thromboembolism prophylaxis after gynecological pelvic cancer surgery with Rivaroxaban versus enoxaparin) trial showed that 10mg of rivaroxaban had a similar outcome to 40mg enoxaparin for thromboembolism prophylaxis after major gynecological cancer surgery [14]. Fondaparinux, a modern antithrombotic agent with pure anti-factor Xa activity, effectively prevents symptomatic and asymptomatic venous thromboembolic events among older acutely ill medical patients aged 60 or above. The ARTEMIS study, which compared the effects of 2.5 mg/day of fondaparinux with a placebo for 6 to 14 days, revealed a substantial reduction in VTE incidence by day 15. Fondaparinux exhibited a 49.5% lower risk of VTE and no fatal PE cases in the treatment group, with similar rates of significant bleeding between the two groups. This study highlights the efficacy of fondaparinux in reducing VTE and fatal PE occurrences in hospitalized patients with acute medical conditions without increasing the risk of significant bleeding [15]. Fondaparinux has also shown benefits in thromboembolism prophylaxis in patients with heparin-induced thrombocytopenia (HIT). However, major bleeding was recorded in patients with creatinine clearance <30ml/min [16].

These trials and analyses strongly endorse using anticoagulants for VTE prophylaxis for at-risk, acutely ill medical patients. This approach effectively lowers the risk of fatal and non-fatal PE and DVT, although there is no significant effect on mortality in medical patients. A meta-analysis conducted by Wein et al. attributed this finding to increased comorbidities in this group of patients [17]. This is in contrast to surgical patients where chemoprophylaxis has shown reduction in mortality [18]. It is also important to note that while the use of anticoagulants for VTE prophylaxis carries a slight increase in bleeding risk compared to a placebo, this risk remains relatively low [3].

Mechanical preventive measures

Graded Compression Stockings

These stockings which can also be referred to as thromboembolic deterrents constitute one of the major mechanical methods of preventing thromboembolic events [19]. It usually comes in various sizes and the major function is to compress the legs, especially the thighs. Aside from being the major mechanical preventive method, it is also very affordable and easy to use [19].

The stockings help in preventing thromboembolic events by altering several pathogenic events in VTE. One of the major ways it is used in reducing the risk of VTE is to compress the leg thereby lowering the cross-sectional area of the vein. Consequently, there is an increase in the velocity across the vessel and this would impede venous stasis. Another way it exerts a favorable effect is to compress the ankle further up thereby increasing the blood flow. Also, TED stockings increase the pumping effect of the calf muscles, and this enhances the way the venous valves work thereby altering the pooling of blood in the veins [19]. If the appropriate compression stockings are applied within 2-3 weeks after diagnosis, the rate of post-thrombotic syndrome is reduced by 50% [20].

Efficacy of TED Stockings

A systematic study done on compression stockings for the prevention of DVT revealed that fewer patients (13%) developed DVT while on stockings compared to patients without stockings (26%) [21]. It was finalized that graduated compression stockings were very useful and efficient in reducing the risk of DV in this study [21].

However, in a trial called the CLOTS trial 1, it was observed that there was a non-significant risk reduction in symptomatic and asymptomatic deep vein thrombosis with graded compression stockings. This trial suggested that further study needs to be done to see if graded compression stocking prevents DVT in both medical and surgical patients [21].

Limitations to the Use of TED Stockings

One major limitation is the application of the wrong size of stocking. This can irritate the skin and cause ulcer formation and sensory loss resulting in neuropathy. Another disadvantage of using a poorly fitted stocking is the fact that it can cause a tourniquet effect. Consequently, this can impede blood flow both in the venous and arterial vessels resulting in venous thrombosis and arterial ischemia [19]. Some major contraindications to the use of TED stockings include diabetic neuropathy, dermatological conditions, and complicated peripheral vascular disease [19].

Intermittent Pneumatic Compression (IPC) Device

An IPC device is a type of mechanical device that is used to prevent VTE. It is designed in the form of a fabric sleeve which is attached to a patient’s leg and fitted to a pump [22]. These components work by ensuring that pumped air is periodically delivered [22]. Also, the pumping of air ensures that deep venous systems are compressed thereby displacing the blood proximally [19]. When the cuffs are deflated, the veins get refilled distally. By so doing, there is a pulsatile stimulation and maintenance of blood flow [19]. In addition, the stimulating effect of the venous walls' fibrinolytic activity helps to reduce VTE by decreasing the plasminogen activator inhibitor level [22].

Limitations to the Use of IPC

The use of the IPC is limited by improper fitting of devices and inaccurate use of devices. Other complications include damage to nerves and blood vessels, compartment syndrome, and compartment syndrome [19].

Efficacy of IPC Stockings

A systematic review and meta-analysis done on the efficacy of intermittent pneumatic compression for VTE prophylaxis in patients undergoing gynecologic surgery showed that Intermittent pneumatic compression devices lowered the risk of deep vein thrombosis compared with control [23].

Intermittent Pneumatic Compression versus Graded Compression Stockings for Thromboprophylaxis

The first meta-analysis done in the year 1998 discovered that intermittent pneumatic compression stockings reduced the relative risk of DVT by 47% when compared to graded compression stockings [19]. Also, it was noted that the relative risk of DVT in both high-risk and low-risk patients was significantly reduced [19]. Based on this, intermittent pneumatic compression was observed to be more effective than graded compression stockings in preventing DVT [19].

However, the risk of bleeding is often considered in the choice between pharmacological and mechanical methods of prophylaxis, Decousus et al. found only three major risk factors at admission for bleeding in medical patients: Active gastroduodenal ulcer, prior bleeding within the last three months, and low platelet count [24]. Any active bleeding and INR greater than 1.5 are also contra-indications to pharmacological prophylaxis. In such cases, IPC which has shown a greater efficacy than GCS, or IVC filters in cases of established DVT, can be used. Pharmacological prophylaxis has been shown to be more effective when combined with mechanical prophylaxis in surgical patients [25].

Multimodal approaches

The multimodal approach has been shown to reduce the incidence of VTE in hospitalized surgical patients, although similar benefits have not been observed in medically ill patients [26]. It includes a combination of mechanical and pharmacological therapy to prevent the occurrence of VTE. The following are scientific evidence to substantiate this:

Kakkos et al., in their research, revealed that the multimodal VTE prophylaxis protocol involving the use of intermittent pneumatic leg compression pumps (a mechanical approach) and pharmacotherapy in patients undergoing surgery or admitted with trauma yielded a very low rate of DVT events with a 2.9% incidence rate versus 6.2% when pharmacological therapy was used alone [27]. Furthermore, the study showed that the use of a combined intermittent pneumatic leg compression pump and pharmacotherapy resulted in a reduced rate of DVT when compared to the use of intermittent pneumatic pump alone (2.19% vs. 4.1%) [27].

Della Valle et al., in their comprehensive study on VTE multimodal prophylaxis protocol, illuminated a reduced incidence of thromboembolic disease of as low as 2.5% symptomatic DVT, 0.6% pulmonary embolism, and 6.4% asymptomatic DVT in a patient who underwent total hip arthroplasty when observed over three months [28]. The multimodal approaches employed in their study were intravenous heparin (pharmacological agent), pneumatic compression (mechanical), knee-high elastic stockings (mechanical), pre-operative discontinuation of procoagulant medications, and early mobilization, among others [28].

Implementation of preventive protocols

VTE continues to pose a substantial burden, representing a significant healthcare issue that results in considerable morbidity, mortality, and resource utilization [29]. Consequently, the implementation of protocols aimed at preventing VTE becomes an absolute necessity. Numerous studies have delved into VTE management, with their discoveries integrated into evidence-based protocols extensively adopted by both the American College of Chest Physicians (ACCP) and the International Union of Angiology [30]. To ensure successful implementation, guidelines play a crucial role by providing clear direction. The current ACCP guidelines specifically focus on acutely ill medical patients who have been admitted due to conditions such as heart failure, severe respiratory disease, or being bedridden, accompanied by one or more additional risk factors like cancer, previous VTE, sepsis, acute neurological disease, or inflammatory bowel disease [30].

In terms of guidelines, the choice of drugs, dosage, duration, and frequency holds great significance. Recommended anticoagulants encompass LMWH, UFH, and the factor Xa inhibitor fondaparinux. These selections were based on extensive randomized clinical trials, demonstrating their safety and effectiveness when compared to a placebo [10].

The dosing type and frequency are pivotal factors in both preventing and reducing VTE-related mortality rates and optimizing hospital resource management. Unfractionated heparin, owing to its relatively short half-life and low bioavailability, is administered subcutaneously two to three times daily [31]. Conversely, LMWH is typically administered once a day due to its longer half-life and higher bioavailability, enhancing convenience [31]. Guidelines should also address the duration of prophylactic VTE management. ACCP and the International Union of Angiology recommend extended-duration prophylaxis for certain high-risk surgical patients with treatment durations ranging from 10-35 days, depending on the severity of surgical intervention [30]. Similar benefits of extended-duration prophylaxis have also been observed in certain medically ill patients including acutely ill medical patients with level 1 immobility, those older than 75 years, and women [32]. 

While guidelines are essential for successful implementation, it is crucial to understand that selecting an anticoagulant isn't a one-size-fits-all approach. Comorbid factors can significantly impact drug choices. For instance, in patients with renal insufficiency, using LMWHs may lead to dose accumulation [31]. Therefore, it is pivotal to make dose adjustments for patients with mild to moderate renal insufficiency. Additionally, certain anticoagulants, like fondaparinux, should be avoided in patients with severe renal insufficiency [31]. 

In implementing protocols and guidelines, clinicians must combine a high level of suspicion with a deep understanding of evidence-based literature and guidelines, crucial for choosing the right drug, its proper dosage, and the necessary duration in VTE prophylaxis for medically ill patients. Despite the potential costs of medications, VTE prophylaxis remains cost-effective, given potential efficacy and safety benefits. Notably, the underutilization of VTE prophylaxis has led to government and regulatory involvement to promote its appropriate use in US hospitals, to improve clinical and economic outcomes for at-risk medical patients [29].

Patient-centered considerations

VTE being a preventable cause of death requires optimal patient compliance and medication adherence. It is therefore extremely important that proper education and counseling be given to the population at risk; emphasizing the role of prevention against facing the gruesome consequences VTE comes with. This is not to say that prevention does not come with its perils. Several studies have shown how several factors illustrate significant variability in patient values and preferences regarding thrombosis prophylaxis and treatment. Some of the factors include the inconvenience of daily treatment, previous experience with prophylaxis, regular testing and monitoring, side effects, adequate education, and overall willingness [33].

Some of these studies involved patients with atrial fibrillation, VTE, stroke or myocardial infarction prophylaxis, on thrombolysis in acute stroke, and strategies used to elicit their preferences include analog scales, standard gamble, time trade-off, probability trade-off technique, decision aids, presentation of hypothetical scenarios during interview or surveys [33]. This study evaluated health state utilities that were obtained from participants about both long-term and short-term outcomes of both thrombolysis and prophylaxis treatment. Each participant's utility value, graded on a scale of 0 to 1 with 0 being equivalent to worse health or disutility, and 1 being optimal health reflects his or her perception of a given health state or outcome [33]. Results from this study inferred that the majority of the participants would rather comply with treatment than face consequences like stroke as they tended to place higher disutility on stroke than treatment burden [33].

VTE is a major cause of maternal morbidity and mortality and pregnancy increases the risk fivefold as mentioned by the CDC [34]. A study evaluated women’s values and preferences on the use of low molecular weight heparin in pregnancy using a mixed-method systematic approach. This was both a quantitative and qualitative review gathered from data from 427 women with a mean age of 33.8 years. The participants were widely varied, from women in the antepartum period where the risk of VTE was exclusively due to a history of VTE, use of unfractionated heparin for both preventive and treatment purposes in pregnant women, to postpartum with multiple risk factors for VTE [35]. Factors tested include the willingness to take LMWH, pregnancy with LMWH prophylaxis, beliefs towards the harm, overuse, necessity, and concerns of taking LMWH, reasons for not being adherent when using LMWH, preference for route of administration, preferred amount of information regarding LMWH, experience, and medication concerns [35].

The study concluded that the majority consider that the benefits of treatment outweigh the daily inconveniences, the main concerns are the safety and injection administration, people who had a much higher risk were more willing to take it and finally women prefer making informed decisions [35]. These studies therefore highlight the importance and role of patient-clinician collaboration in VTE prophylaxis, as patients tend to do better when provided adequate information and support with informed decision making. Optimal prevention can only be achieved with medication adherence and patient compliance.

Challenges in implementation of VTE prevention guidelines

Translating guidelines for VTE into practical clinical applications faces considerable challenges, despite their growing popularity, primarily due to various barriers [36]. These obstacles encompass factors such as limited awareness of the guidelines, resistance to change among healthcare professionals, doubts regarding the effectiveness of the recommendations, concerns about potential side effects, difficulties in recalling the guidelines, the absence of institutional policies reinforcing these recommendations, and economic constraints. Consequently, the adoption of new recommendations can often be a prolonged process, sometimes spanning several years or even decades before effective therapies become standard practice after their initial publication in case reports [36].

To surmount these hurdles and ensure the successful implementation of new medical recommendations, it is imperative to adopt a comprehensive strategy. This strategy should involve the development of user-friendly algorithms, the creation of continuous medical education materials and lectures, the utilization of electronic or paper-based alerts, the provision of tools to streamline the evaluation and prescription processes, and the regular conduct of audits to demonstrate results to healthcare practitioners. Furthermore, gaining the endorsement of medical societies related to VTE, establishing multidisciplinary committees within each institution, and engaging opinion leaders known for their proactive and steadfast attitudes are essential elements contributing to the program's success [37].

Another effective approach to improve the implementation of medical guidelines, particularly in the context of VTE prevention, is through patient education. A notable observation is that approximately 15% of clinician-ordered doses of injectable pharmacological prophylaxis to prevent VTE go unadministered, with patient refusal accounting for nearly 50% of these omitted doses [38]. A study conducted in 2004 revealed that a pharmacist-led individualized patient education program led to significantly higher medication adherence for clinician-ordered injectable pharmacological VTE prophylaxis. This program was associated with a one-third lower frequency of patient refusal as a reason for omitted doses of clinician-ordered injectable prophylactic anticoagulation. Notably, the pharmacist-led patient education program proved feasible, with 99% of sessions completed within 24 hours of the initial order for prophylactic anticoagulation [38].

## Conclusions

VTE remains a common complication among hospitalized patients despite it being largely preventable. Preventing VTE via risk identification and thromboprophylaxis via mechanical, pharmaceutical methods, or a multimodal approach in a patient-centered manner can significantly alleviate the associated morbidity, mortality, and financial strain on patients. We strongly urge healthcare professionals to diligently follow established VTE guidelines, and we call upon policymakers to facilitate the development of user-friendly algorithms. Additionally, we emphasize the importance of patient education to encourage cooperation and adherence to VTE prevention strategies.
